# Ultrasmall Gold Nanoparticles Boost Radiotherapy and Protect against Radiation Damage

**DOI:** 10.1021/acsomega.5c05201

**Published:** 2025-09-02

**Authors:** Marina Piacenti-Silva, Hulder Henrique Zaparoli, Mileni Mayumi Isikawa, Eder José Guidelli, Eric Crampon, Carolina Letícia Zilli Vieira

**Affiliations:** † 153994 São Paulo State University, School of Sciences, Bauru Campus, Bauru, SP BR 17033-360, Brazil; ‡ University of São Paulo, School of Philosophy, Sciences and Letters at Ribeirão Preto, Ribeirão Preto, SP BR 14040-900, Brazil; ∥ 487038 Analytical Development at Takeda Vaccines Business Unit, Cambridge, Massachusetts 02139, United States; ⊥ Department of Environmental Health, Harvard T. H. Chan School of Public Health, Boston, Massachusetts 02115, United States

## Abstract

This study investigates the effects of ultrasmall (∼4 nm) gold nanoparticles (AuNPs) combined with X-ray irradiation to enhance radiotherapy efficacy. Using the in vivo *Drosophila melanogaster* model, we observed that while AuNPs alone delayed embryonic development, their combination with irradiation completely halted it. Lifespan analysis showed that irradiated flies fed with AuNPs had a slight survival advantage, suggesting a protective effect against radiation-induced oxidative stress. Immunofluorescence analysis revealed increased DNA damage (or repair) in the flies, supporting the potential of AuNPs to boost the local radiation dose and offer protection against radiation-induced damage, with implications for optimized therapeutic strategies.

## Graphical abstract

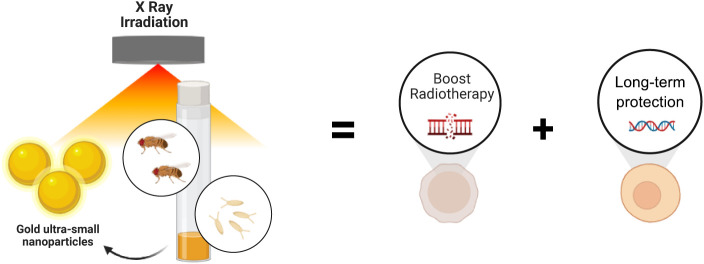

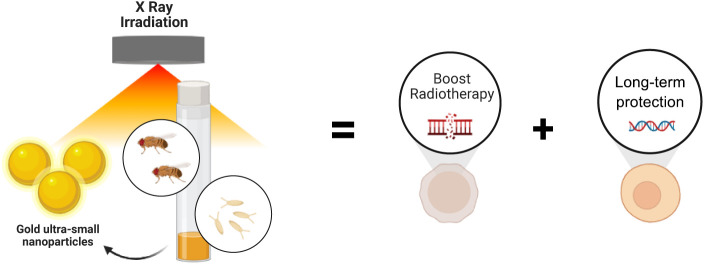

## 1 Introduction

Radiotherapy aims to maximize the lethal dose to tumor cells while minimizing damage to healthy tissues.^1^ However, tumor cells often exhibit intrinsic resistance to radiation, limiting its effectiveness. Recently, gold nanoparticles (AuNPs) have emerged as a strategy to enhance radiosensitization due to their strong surface plasmon resonance (SPR) properties and high atomic number, which increase radiation interaction and the production of free radicals that damage tumor DNA.^2,3^

AuNPs can significantly enhance energy deposition depending on their size, shape, and distribution.^4,5^ Different geometries and sizes of AuNPs have been studied to optimize dose delivery in tissues, improving radiotherapy efficacy while minimizing dose to surrounding healthy tissues. The internalization of AuNPs by tumor cells allows them to accumulate near the nucleus, facilitating DNA damage upon irradiation.^6^ However, clinical application faces challenges such as optimizing biodistribution, biocompatibility, tumor targeting specificity, and mitigating potential toxic effects.^7^

The size of AuNPs influences their toxicity and therapeutic efficacy, with smaller particles showing greater reactivity and the potential to cross biological barriers more easily, increasing both efficacy and toxicity risks due to inflammation and oxidative stress.^8,9^ The optimal size of AuNPs for radiosensitization, without increasing systemic toxicity, remains under investigation.^10,11^

Importantly, despite their anatomical differences, *Drosophila melanogaster* and humans share highly conserved molecular responses to ionizing radiation. Radiobiology is based on the ability of cells to detect and respond to DNA damage to preserve genomic integrity and maintain tissue homeostasis. These responses include cell cycle arrest, activation of DNA repair pathways, and apoptosis, which eliminates damaged cells.^12^ Whether a cell survives or dies is context-dependent and influenced by cell type, developmental stage, and proliferation status. The apoptotic response is particularly relevant in cancer therapy because its dysregulation can lead to tumor resistance or sensitivity to radiotherapy.^13^

The *Drosophila* model has been instrumental in elucidating the molecular and cellular mechanisms activated by genotoxic stress.^14^ Core pathways involved in DNA damage recognition and repair, oxidative stress response, and apoptosis via p53 are evolutionarily conserved across species.^14,15^ Furthermore, *Drosophila* exhibits phenomena such as radiation-induced hormesis and a radioadaptive response that depend on the integrity of autophagy, stress response, and DNA repair pathways.^16^ Interestingly, both *Drosophila* embryos and human organ-specific cells use similar protective strategies to counteract radiation-induced oxidative stress. These strategies include the detoxification of reactive oxygen species (ROS) through antioxidant enzymes, such as superoxide dismutase and catalase, and the activation of DNA damage response pathways. These parallels further strengthen the relevance of Drosophila models in translational radiation research and reinforce the validity and cost-effectiveness of using *Drosophila* as an in vivo model to investigate the biological impact of radiation and nanoparticle exposure.^17,18^

Given these challenges, this study focuses on the use of ultrasmall AuNPs to enhance the local radiation dose in radiotherapy and assess their potential toxic effects and impacts of radiation after nanoparticle administration. Using *Drosophila melanogaster* as a model, we hypothesize that increased local radiation dose could affect pupal hatching, fly longevity, and DNA damage days after exposure.

## 2 Materials and Methods

Our experimental workflow (Figure 1) illustrates the key steps of this study, from nanoparticle synthesis and characterization to biological outcome measurements in *Drosophila melanogaster*, including lifespan, locomotion, and DNA damage assays.

**Figure 1:**
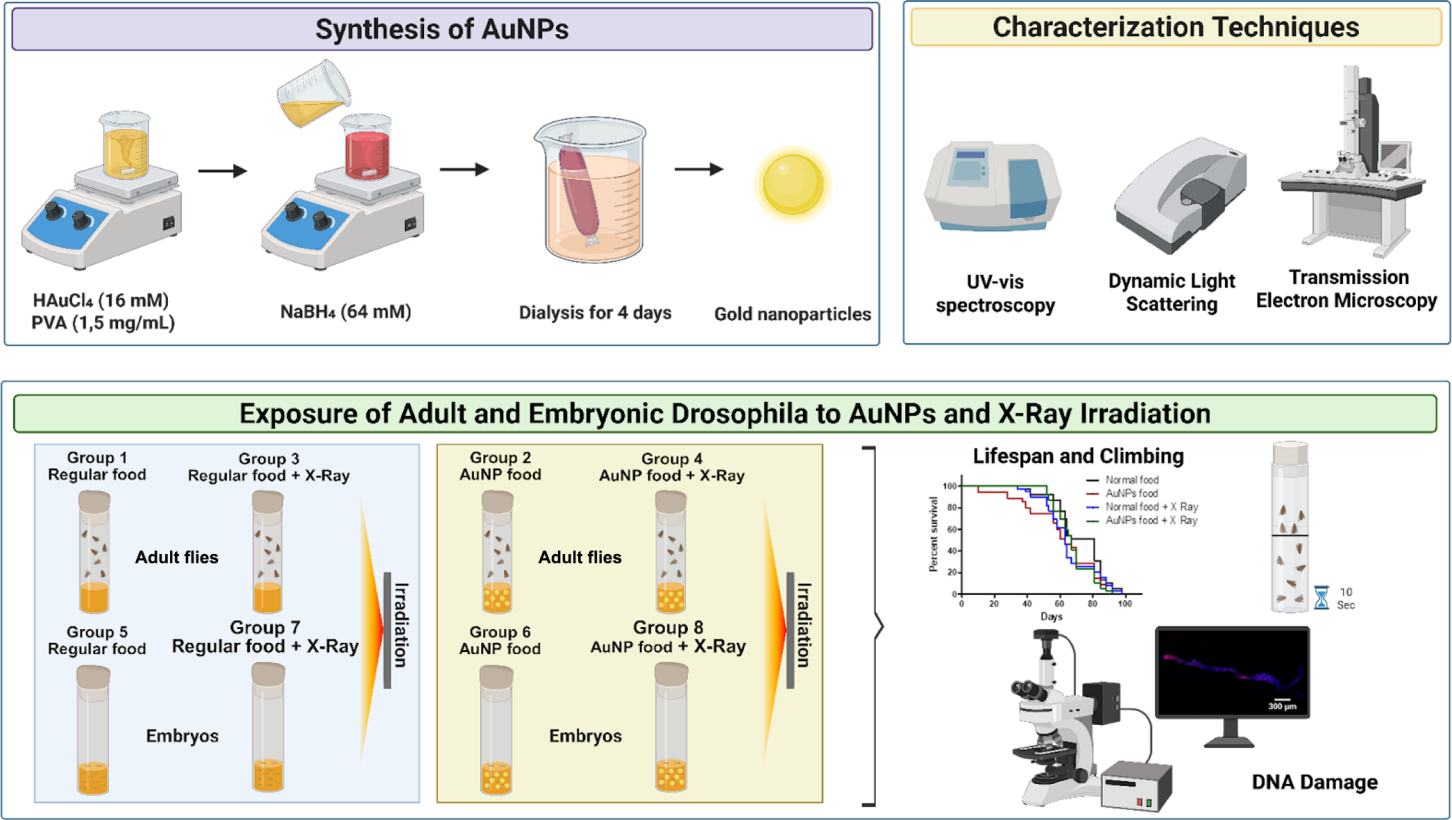
Experimental workflow. AuNPs were synthesized, characterized, and administered to *Drosophila* via food. Flies were or were not exposed to X-rays. The outcomes included lifespan, climbing ability, and DNA damage (γ-H2AV). Created in BioRender. Bauru, G. (2025) https://BioRender.com/e66bvrb.

### 2.1 Gold Nanoparticles – Synthesis and Characterization

AuNPs were synthesized using the chemical reduction method. A solution of HAuCl_4_ (16 mM) and PVA (1.5 mg/mL) was added to a fresh NaBH_4_ (64 mM) solution under vigorous stirring for 12 h. The resulting AuNP colloidal dispersion was dialyzed in 5 L of Milli-Q water for 4 days, with daily water changes to remove reaction residues.

The AuNP plasmon band was confirmed by UV–vis spectroscopy using an Ultrospec 2100 pro (Amersham Pharmacia). Particle size distribution was measured by dynamic light scattering (DLS) using the Zeta Sizer system (Malvern Instruments, U.K.), with a fixed angle (173°) and a 633 nm He–Ne laser. The morphology and size of the AuNPs were examined using transmission electron microscopy (TEM) with a JEOL-JEM-100 CXII (JEOL, Japan). A 1000-fold diluted AuNP sample was dropped onto a copper grid covered with a conductive polymer, and images were processed using ImageJ software.

### 2.2 Flies Strains and AuNPs Exposition

Experiments were conducted using *Drosophila melanogaster* Canton S flies (generously provided by Dr. Dragana Rogulja, Department of Neurosciences, Harvard Medical School), maintained on cornmeal-agar medium under 12 h light/12 h dark cycles (12:12 LD). After embryo synchronization, newly hatched adult flies were collected and mated for 2 days.^19^

Eight experimental groups were formed distributed as follows. Group 1: flies received a standard diet; Group 2: flies received a diet containing AuNPs; Group 3 and Group 4: similar to the first and second groups, respectively, but were also exposed to irradiation; Group 5: embryos received a standard diet; Group 6: embryos received a diet containing AuNPs; Group 7 and Group 8: similar to the seventh and eighth groups, respectively, but were also exposed to irradiation. For each experimental group, around 20 males flies with 3 days old were placed in a vial, with each treatment replicated in triplicate.

The AuNP-enriched diet was prepared by mixing the nanoparticle solution into freshly melted fly food, followed by vigorous stirring before distribution into vials. The final AuNP concentration was set at 100 pmol/L, a nonlethal dose,^20–24^ equivalent to 197 × 10^–7^ mg Au/L or 16 pg/g Au in food. Considering a daily intake of 1.5 μL per fly,^22^ the maximum estimated AuNP dose was 0.03 pg Au/day.

Groups 2, 4, 6, and 8 were fed the AuNP diet until day 11, when irradiation was performed. After exposure, these groups were switched to the standard diet. This design allowed evaluation of both isolated and combined effects of AuNPs and irradiation.

### 2.3 Irradiation

Irradiation was performed using an X-ray source operating at 220 kV and 13 mA, delivering a total dose of 30 Gy in a single fraction at a rate of 3.6 Gy/min. This dose was selected based on previous studies demonstrating its effectiveness in analyzing sublethal biological effects and stress responses induced by irradiation.^25–29^ Immediately after exposure, all flies were transferred to fresh vials containing standard food and maintained in the incubator. The biological effects of AuNPs combined with X-ray irradiation were assessed through developmental timing, pupation, lifespan, and DNA damage via immunofluorescence analysis.

### 2.4 Development and Lifespan

Embryo-containing vials were kept in the incubator, and developmental timing was assessed. Embryo development was quantified based on two parameters: the hatch rate during the first 3 days and the total hatch rate. These metrics represent, respectively, the proportion of flies hatched within the first 3 days relative to the total number of hatched flies, and the total number of hatched flies relative to the total number of pupae.^30–32^

For lifespan analysis, adult flies were transferred to fresh vials every 3 to 4 days. Importantly, transfers were conducted without CO_2_ anesthesia to avoid stress that could influence longevity.^19^ The number of dead flies was recorded at each transfer until all individuals had died. This method allows for continuous and accurate monitoring of mortality over time, enabling reliable statistical comparisons of lifespan across treatment groups. The decision to avoid CO_2_ and the transfer frequency followed best practices described in the literature to minimize experimental bias.^19^

### 2.5 DNA Damage

To evaluate the local effects of irradiation with and without AuNPs, adult flies were anesthetized 3 days postirradiation, and gut tissues were dissected in 1X phosphate-buffered saline (PBS). Guts were fixed in 4% paraformaldehyde (PFA) for 1 h on a shaker at room temperature. After three 20 min PBS washes, samples were incubated overnight at 4 °C in PBS containing 0.5% Triton X-100 and 2% bovine serum albumin (BSA).

Immunostaining was performed using the primary antibody mouse anti-γH2Av (1:40, DSHB), incubated for ∼24 h at 4 °C in PBS/0.5% Triton X-100 + 2% BSA. Samples were then washed three times (20 min each) in PBS/0.5% Triton X-100 and incubated for 2 h at room temperature with secondary antibody Alexa Fluor 568 donkey antimouse and Hoechst dye (Invitrogen Molecular Probes, 1:1000), also diluted in PBS/0.5% Triton X-100 + 2% BSA. Final washes (3 × 20 min) were performed in PBS. Tissues were mounted between glass slides and coverslips (Electron Microscopy Sciences) using Prolong Gold Antifade mounting medium (Invitrogen).^33^

Images were acquired with a Leica SP8 confocal microscope. Laser, filter, and gain settings were kept constant across experimental groups. All samples were imaged sequentially using a 20× oil immersion objective and analyzed with Fiji software.

### 2.6 Statistical Analysis

All statistical analyses were performed using RStudio software. Group comparisons of embryo hatch rates were conducted using the Mann–Whitney test. Survival distributions from lifespan experiments were evaluated using bootstrap analysis of median survival, with significance adjusted for multiple comparisons using Bonferroni’s method.^33^ Quantification of immunofluorescence images was carried out using Fiji/ImageJ software.

## 3 Results

TEM analysis (Figure 2a) revealed the formation of ultrasmall, monodisperse gold nanoparticles with spherical morphology. The particle size distribution obtained from TEM images (*n* = 100) was consistent with the hydrodynamic diameter measured by DLS (Figure 2b), with average sizes ranging from 4 to 5 nm. UV–vis spectroscopy (Figure 2c) showed a characteristic plasmon band between 500–550 nm, confirming the presence of spherical AuNPs. These results demonstrate the successful synthesis of well-defined, ultrasmall gold nanoparticles.

**Figure 2:**
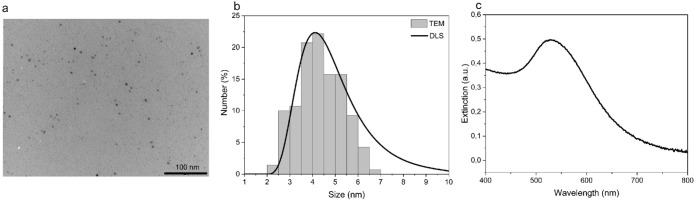
(a) TEM images of the gold nanoparticles after dialysis. (b) Particle size distribution (diameter) obtained from TEM and DLS. (c) UV–vis absorption spectrum of gold nanoparticles depicting the plasmon band characteristic of spherical gold nanoparticles.

### 3.1 Development and Lifespan

Following irradiation, embryo-containing vials were incubated and the eclosion rate was monitored. Ten days after exposure, pupae from irradiated and nonirradiated embryos maintained on either standard food or AuNP-supplemented food were evaluated (Figure 3a and b). Three days after the onset of hatching, the number of emerged flies and the total number of hatched pupae were recorded (Figure 3c). In addition, survival was further analyzed by Kaplan–Meier curves to compare differences among groups (Figure 3d).

**Figure 3:**
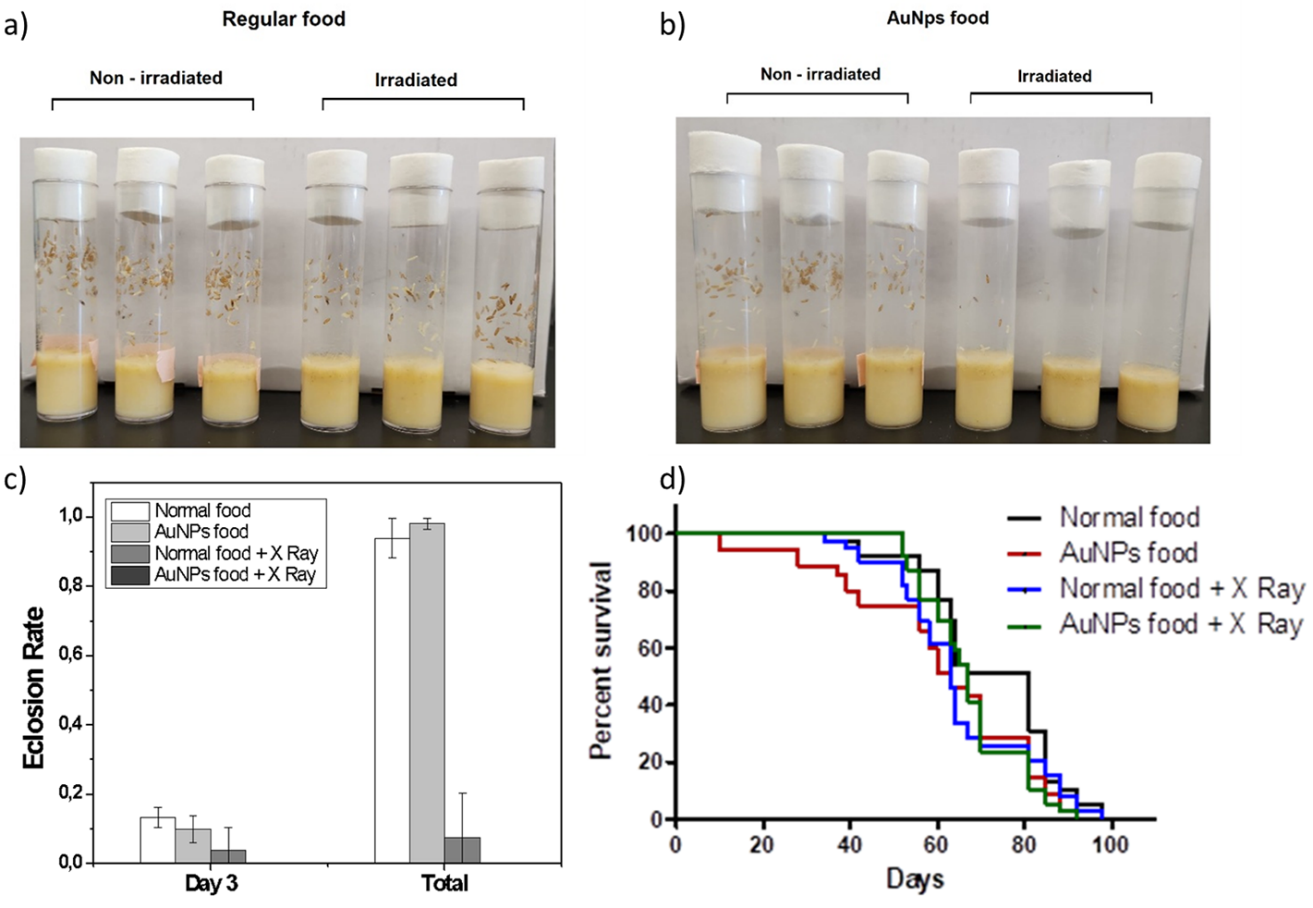
Vials containing eggs, larvae and pupae in a) regular diet and b) AuNPs diet (photos taken by the author MPS); c) hatch rate at day 3 and total number of pupae hatched; d) Kaplan–Meier survival curves of adult flies.

The Mann–Whitney test revealed no significant differences in the number of hatched flies between irradiated and nonirradiated groups under standard diet conditions, either during the first 3 days or in total pupal eclosion. However, under the AuNP-supplemented diet, although pupae were observed in the irradiated group, none reached adulthood (Figure 3b). While formal statistical analysis was not applicable in this case, the complete absence of adult emergence strongly indicates that the combination of AuNPs and irradiation had a synergistically deleterious effect on embryo development.

Kaplan–Meier survival curves (Figure 3d) showed that, as expected, *Canton S* flies displayed extended lifespans, reaching up to 98 days.^34^ Statistical comparisons revealed significant differences among all treatment groups. Irradiation consistently reduced survival compared to nonirradiated groups, regardless of diet. Although some irradiated flies survived to approximately 100 days, the majority died around day 70. Interestingly, irradiated flies fed with AuNPs exhibited a modest increase in survival relative to irradiated flies on the standard diet, suggesting a slight protective effect against irradiation-induced stress.

### 3.2 DNA Damage

To evaluate the cellular effects observed with the combination of AuNPs and radiation, immunofluorescence images were obtained from the guts of flies from different groups (Figure 4). In blue, one can see the labeling of the Hoechst dye, which binds to cellular DNA and highlights the morphology and nuclear density of the cells in each condition. In red, γ-H2AV labeling identifies sites of active DNA damage or repair.

**Figure 4:**
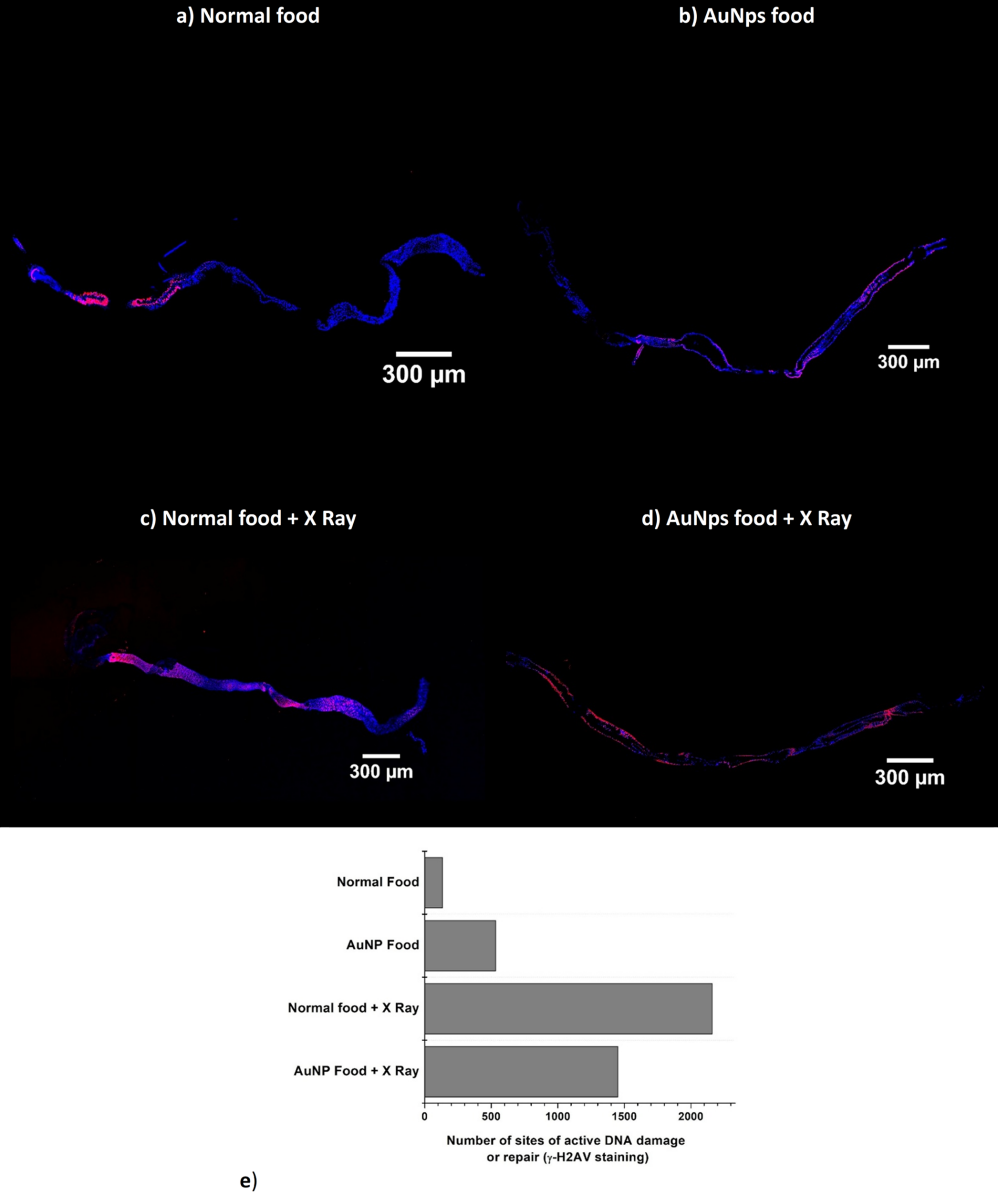
Immunofluorescence images from guts of flies from different groups. a) Normal food; b) AuNPs food; c) Normal food + X Ray; d) AuNPs food + X Ray. In blue labeling of the Hoechst dye and in red, γ-H2AV labeling identifies sites of active DNA damage or repair. e) Number of cells stained for γ-H2AV.

Qualitative analysis of γ-H2AV immunostaining revealed increased DNA damage in nonirradiated flies fed with AuNPs compared to the control group on a standard diet, suggesting that AuNP exposure alone induces DNA damage. Among irradiated flies, those on a standard diet exhibited greater γ-H2AV labeling than the control group, while flies exposed to both AuNPs and X-rays showed the highest overall labeling intensity.

Quantitative analysis of γ-H2AV-positive cells (Figure 4e) confirmed that the control group (standard diet, no irradiation) had the lowest level of DNA damage. Flies fed with AuNPs alone exhibited a moderate increase in damage, while the irradiated group on a standard diet showed a more pronounced response. Notably, the group exposed to both AuNPs and irradiation demonstrated a slightly lower number of γ-H2AV-positive cells than the irradiated group without AuNPs, suggesting that AuNPs may influence not only damage induction but also cellular repair responses.

## 4 Discussion

This study used *Drosophila melanogaster* as a model organism to gain valuable insight into fundamental biological responses to radiation and nanoparticle exposure that are highly relevant to mammalian systems. Although *Drosophila* differ morphologically from humans, they have long been established as robust in vivo models due to their highly conserved molecular pathways.^17^ Key processes, such as DNA damage signaling, repair mechanisms, the oxidative stress response, and cell death regulation, are functionally comparable to those in mammals.^27,28^ Additionally, *Drosophila* offers genetic tractability, a rapid generation time, and the ability to conduct cost-effective, large-scale testing, making it a powerful platform for evaluating nanoparticle-radiation interactions.^18^

Previous studies have used *Drosophila* successfully to assess radiation-induced gut damage, DNA repair dynamics, and the effects of metal-based nanoparticles.^25^ These studies support the translational relevance of our findings. Although *Drosophila* lacks the complex tissue architecture of mammals, its cellular responses closely correspond to essential pathways observed in complex organisms. Thus, the findings presented herein regarding enhanced DNA damage, lifespan modulation, and developmental arrest lay a valuable groundwork for future translational studies in mammalian models of radiotherapy.

Based on this rationale, we investigated the combined effects of ultrasmall gold nanoparticles (AuNPs) and X-ray irradiation on the development and lifespan of *Drosophila melanogaster*. We synthesized AuNPs with an average diameter of ∼4 nm and characterized them using transmission electron microscopy (TEM), dynamic light scattering (DLS), and UV–visible spectroscopy. The AuNPs exhibited a plasmonic peak at 534 nm, confirming their potential to enhance local radiation dose deposition. We evaluated biological responses through embryonic development, adult lifespan, and DNA damage using confocal microscopy and γ-H2AV immunostaining.

Because of their small size, AuNPs can more effectively penetrate biological tissues and interact closely with cellular components. This supports their role as efficient radiosensitizers without substantially increasing systemic toxicity. Although we did not directly assess AuNP internalization in *Drosophila*, previous studies have demonstrated nanoparticle uptake in fly tissues such as the fat body. These studies have also shown that nanoparticles accumulate in lipid droplets and activate PI3K/Akt/mTOR signaling without inducing stress responses.^21^ Together, TEM and ICP-MS analyses in mammalian cells confirm the cytoplasmic accumulation of AuNPs near the nucleus, which is essential for efficient radiosensitization.^35^

Exposure to AuNPs alone resulted in developmental delays, though it had no significant impact on hatching rates. However, when combined with irradiation, embryonic development was completely halted, suggesting a deleterious synergistic effect. This finding is consistent with previous reports indicating that nanoparticles can amplify the biological impact of radiation and enhance tumor targeting in clinical radiotherapy.^28^

Lifespan analysis revealed that irradiation reduced overall survival. However, flies that received both AuNPs and irradiation showed a slight survival advantage compared to those that received irradiation alone. This finding suggests a potential protective effect that may be due to the reduction of oxidative stress. Additionally, DNA damage analysis revealed an increase in γ-H2AV foci in flies exposed to both AuNPs and X-rays. This lends further support to the hypothesis that, while enhancing the effects of radiation, AuNPs may also modulate cellular repair mechanisms.

These biological effects are consistent with the anticipated changes due to the radiation quality induced by high-Z nanoparticles. The presence of AuNPs has been shown to modify the local radiation field by emitting secondary electrons, including photoelectrons, Compton electrons, and Auger electrons, when exposed to ionizing radiation.^36–38^ Low-energy electrons deposit energy within nanometric ranges, leading to two phenomena: highly localized ionization events and increased production of reactive oxygen species (ROS). Furthermore, these events result in DNA damage clusters, which are particularly challenging to repair and often more biologically lethal than isolated DNA breaks.^39^ The γ-H2AV accumulation observed in this study supports this mechanism, suggesting that AuNPs potentiate both oxidative and direct DNA damage triggered by X-ray exposure.^2,40,41^

In nonirradiated flies, our findings are consistent with previous studies showing that AuNPs can induce DNA fragmentation and shorten lifespan.^20,22,42^ However, there is also some evidence supporting a dual role for AuNPs as radioprotective agents that could improve tissue resilience and therapeutic outcomes.^43,44^

Beyond their physical properties that enhance local radiation dose through secondary electron emission, ultrasmall AuNPs may modulate biological pathways associated with oxidative stress and DNA repair. Prior studies have demonstrated that AuNPs can alter the redox balance of cells,^45^ reduce ROS accumulation,^46^ and activate antioxidant defenses via the Nrf2 pathway,^47,48^ which may partly explain the mild protective effects observed in our lifespan assays.

The localized generation of low-energy Auger electrons leads to clustered DNA damage that is difficult to repair, thereby increasing the cytotoxicity of radiotherapy.^35^ Furthermore, AuNPs may interfere with DNA repair signaling by affecting the activity of key proteins involved in the ATM/ATR and p53 pathways, depending on their intracellular localization and radiation context.^49,50^ Huang et al. (2024)^51^ demonstrated that antiradiation nanoparticles composed of folinic acid and ferulic acid protected immune organs from radiation-induced damage by suppressing the IKK/IκB/NF-κB signaling pathway. These findings highlight the potential of multifunctional nanoparticles not only to enhance radiotherapeutic efficacy but also to preserve healthy tissue integrity. While our study did not assess immunological parameters, emerging evidence supports a dual role for AuNPs as both radiosensitizers and immunomodulators.^52^

Collectively, our findings establish *Drosophila melanogaster* as a viable and informative in vivo model for preliminary evaluation of nanoparticle-enhanced radiotherapy, while underscoring the need for further validation in mammalian systems to ensure translational applicability.

## 5 Conclusion

In conclusion, our results demonstrate that the combination of ultrasmall AuNPs and X-ray irradiation significantly impacts embryonic development, adult lifespan, and DNA integrity in *Drosophila melanogaster*. Not only do AuNPs enhance radiation-induced effects, they may also play a role in modulating cellular stress responses. These findings suggest the potential of AuNPs as radiosensitizers and protective agents in radiotherapy. However, due to the differences in development between *Drosophila* embryos and human organ systems, more research is needed in mammalian models to validate these observations and explore their clinical applicability.

## 6 Limitations and Future Directions

The findings of this study provide important insights into the modulation of radiobiological responses by nanoparticles. However, several aspects require further investigation. First, only a single radiation dose of 30 Gy was assessed. This dose was selected based on prior studies that demonstrated sublethal biological effects in Drosophila models. Exploring additional dose points would allow for the creation of a more comprehensive dose–response curve. The concentrations of AuNPs used in this study (100 pmol/L) were chosen based on preceding studies indicating minimal toxicity. However, employing varying concentrations could facilitate a more profound understanding of the nanoparticle burden and its biological ramifications. Furthermore, the present study focused exclusively on ultrasmall AuNPs (∼4 nm), which behave differently from larger nanoparticles with regard to cellular uptake and radiosensitization. Comparative analyses involving a range of particle sizes and compositions would contribute to a more robust understanding of the mechanisms involved.

Finally, although using *Drosophila melanogaster* provides valuable insights into conserved radiobiological mechanisms, it is important to acknowledge the limitations of extrapolating across species. Differences in tissue architecture, metabolic rates, and developmental biology between flies and mammals may affect the progression of radiation-induced damage. Therefore, future studies using mammalian models are necessary to validate the effects observed with AuNPs and confirm their therapeutic relevance in a clinical context.

## Data Availability

All data generated or analyzed during this study are included in this published article.

## Abbreviations

AuNPs: Gold Nanoparticles
BSA: Bovine Serum Albumin
CO_2_: Carbon Dioxide
DLS: Dynamic Light Scattering
DNA: Deoxyribonucleic Acid
DSHB: Developmental Studies Hybridoma Bank
LD: Light/Dark (cycle)
PBS: Phosphate-Buffered Saline
PFA: Paraformaldehyde
SPR: Surface Plasmon Resonance
TEM: Transmission Electron Microscopy
UV–vis: Ultraviolet–Visible Spectroscopy
γ-H2AV: Phosphorylated H2A Variant (marker of DNA damage)
